# Relapsed Acute Myeloid Leukemia With Early Presentation As Leukemia Cutis, Refractory to Second-Line Treatment

**DOI:** 10.7759/cureus.80114

**Published:** 2025-03-05

**Authors:** Emin Gayibov, Aditi Karambelkar, Virushnee Senthilkumar, Amita J Dsouza, Amit K Correa

**Affiliations:** 1 Third Faculty of Medicine, Charles University, Prague, CZE; 2 Department of Surgery, University Hospital Královské Vinohrady, Prague, CZE; 3 Department of Medicine, Medical College Baroda, Vadodara, IND; 4 Department of Medicine, Coimbatore Medical College and Hospital, Coimbatore, IND; 5 Department of Hematology and Oncology, Hospital Corporation of America (HCA) Houston Healthcare Clear Lake, Houston, USA; 6 Department of Hematology and Oncology, University of Texas Health Science Center at Houston, Houston, USA; 7 Department of Hematology and Oncology, Memorial Hermann Cancer Center - Texas Medical Center, Houston, USA; 8 Department of Hematology and Oncology, University of Texas (UT) Physicians Comprehensive Adult Sickle Cell Center, Houston, USA

**Keywords:** acute myeloid leukemia, hematologic neoplasms, leukemia cutis, refractory acute myeloid leukemia, refractory leukemia cutis

## Abstract

Acute myeloid leukemia (AML) is a hematologic malignancy with a poor prognosis, often presenting with systemic and extramedullary manifestations. Leukemia cutis (LC), the infiltration of leukemic cells into the skin, is a rare and clinically significant complication of AML. It often signals extramedullary disease involvement, may precede systemic relapse, and is associated with poor survival outcomes. We report the case of a 36-year-old female with AML who developed recurrent, reddish-brown nodular skin lesions on the face, trunk, and extremities, consistent with LC. Despite achieving initial remission with induction and consolidation chemotherapy, she experienced early LC recurrence, pancytopenia, hepatobiliary dysfunction, and infectious complications, underscoring the aggressive nature of her disease progression. Disease progression was confirmed by bone marrow evaluation, which revealed 60% blasts. Despite intensive chemotherapy, antimicrobial prophylaxis, and granulocyte colony-stimulating factor support, the patient’s condition deteriorated due to refractory disease. This case suggests that LC may serve as a possible early marker of AML relapse, underscoring the importance of timely recognition and intervention. Multidisciplinary management and early diagnostic reassessment are essential to improving outcomes in such patients. Further research is needed to explore novel treatment strategies for AML patients with LC, aiming to enhance survival and disease control.

## Introduction

Acute myeloid leukemia (AML) is a hematological malignancy characterized by the abnormal and uncontrolled proliferation and differentiation of myeloid progenitor cells in the bone marrow, leading to a poor prognosis [1]. In 2024, approximately 20,800 new cases of AML were diagnosed in both sexes, with an estimated 11,220 deaths [2]. The five-year survival rate from 2014 to 2020 was 31.9% [2]. AML primarily affects individuals aged 60 years and older [3]. This condition is often complicated by serious manifestations, including leukostasis, sepsis, tumor lysis syndrome, and disseminated intravascular coagulation, which can lead to severe morbidity and mortality. These complications result from the rapid accumulation of leukemic cells, overwhelming infections, the release of intracellular contents during tumor cell death, and abnormal blood clotting, respectively [4].

Extramedullary involvement in AML represents a critical yet often underrecognized aspect of disease progression. Manifestations outside the bone marrow occur when leukemic cells infiltrate tissues such as the skin, central nervous system, lymph nodes, and visceral organs [5]. These presentations may precede, coincide with, or follow systemic relapse and are associated with worse clinical outcomes [6]. The pathophysiology of extramedullary disease in AML is not fully understood but is thought to involve aberrant expression of adhesion molecules and chemokine receptors that facilitate leukemic cell migration and tissue infiltration [7]. Risk factors for extramedullary involvement include monocytic lineage differentiation, specific cytogenetic abnormalities, and molecular mutations such as KMT2A rearrangements [8].

One of the rare complications of extramedullary involvement in AML is leukemia cutis (LC), a dermatological manifestation characterized by the infiltration of neoplastic leukocytes into the skin. This infiltration results in various skin lesions, such as papules, nodules, macules, and plaques [9,10]. The prevalence of LC among AML patients is estimated to range between 10% and 15%, with certain subtypes, such as monocytic and myelomonocytic AML, demonstrating a higher propensity for skin involvement [11]. The presence of LC is associated with a poor prognosis, particularly when it is the initial presenting symptom of leukemia [11]. Although rare, the presentation and epidemiology of LC in AML underscore the importance of thorough clinical and histopathological evaluation for accurate diagnosis and effective management.

This case report details the clinical course of a 36-year-old female with AML complicated by LC, highlighting the challenges in managing this aggressive complication and emphasizing the importance of early recognition and prompt treatment. Notably, this case is distinguished by early LC recurrence and rapid disease progression despite treatment. The patient’s disease course was multifaceted, involving recurrent cutaneous lesions, pancytopenia, infectious complications, and hepatobiliary abnormalities. Of particular significance, her cutaneous manifestations preceded systemic relapse, illustrating the need for vigilant monitoring and timely diagnostic interventions in AML patients with LC. This case also underscores the critical role of integrating a team-based approach, in other words, involving specialists from different fields to optimize outcomes in patients with complex hematologic malignancies.

## Case presentation

**Figure 1:**
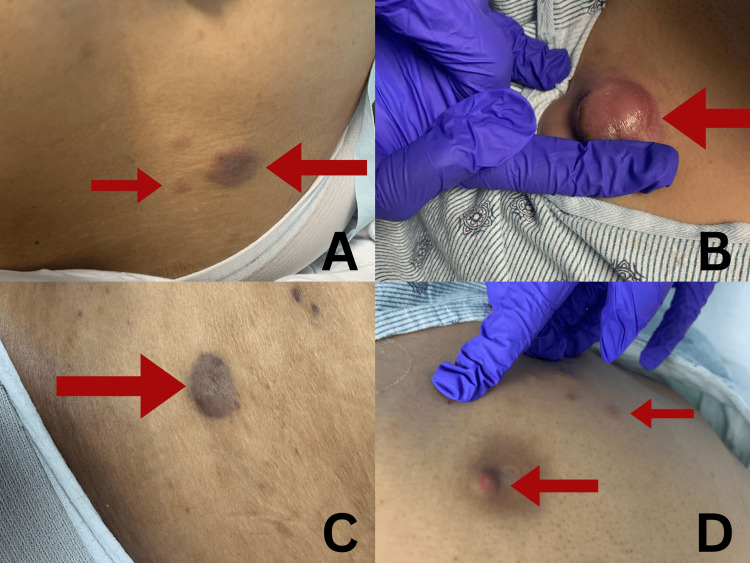
Cutaneous lesions at presentation. The lesions were numerous, scattered, painless, reddish-brown nodules with firm texture. Their sizes ranged from 3 to 5 cm. A finger was placed alongside the nodules to provide a visual scale for size estimation. The lesions have been monitored for changes in size and number, which are indicative of disease progression. (A) Multiple erythematous nodules on the right lower back. (B) A large, tender, violaceous nodule on the right upper chest. (C) A well-defined purplish plaque on the left middle back. (D) Small papulonodular lesions on the left hypochondrium.

This case describes a 36-year-old female with a known diagnosis of AML complicated by LC. The patient's clinical course was complex, characterized by recurrent cutaneous lesions, systemic complications, and significant therapeutic challenges. The patient had a history of recurrent cutaneous lesions affecting her face, back, and legs. At presentation, the skin lesions were numerous, scattered, painless, reddish-brown nodules with a firm texture. Their sizes ranged between 3 and 5 cm, as illustrated in Figures 1A-1D. No significant family history was documented. She denied tobacco, alcohol, or drug use and had no known allergies. She had previously been on prophylactic regimens in consideration of her chemotherapeutic treatment, including valacyclovir, ciprofloxacin, posaconazole, and famotidine.

The patient's AML was initially treated with the standard 7+3 induction regimen, consisting of seven days of continuous-infusion cytarabine and three days of the anthracycline-class agent idarubicin. This was followed by consolidation with four cycles of high-dose cytarabine. Subsequent bone marrow biopsy revealed 4% bone marrow blasts with minimal residual disease (MRD) positive status. Concurrently, skin biopsy findings confirmed LC. She transitioned to a treatment incorporating a regimen of intensive chemotherapy consisting of venetoclax with cladribine, idarubicin, and cytarabine. A repeat bone marrow biopsy revealed a significant reduction in disease burden, showing 3% blasts and MRD-negative status.

Despite initial therapeutic success, the patient's clinical course was complicated by refractory LC in her trunk and extremities, pancytopenia with neutropenic fever, and systemic infections. Blood cultures revealed carbapenem-resistant and extended-spectrum beta-lactamase-producing *Enterobacterales* species. Given its efficacy against multidrug-resistant organisms, cefiderocol was initiated, leading to clinical stabilization. *Streptococcus mitis* was subsequently isolated, likely due to periprocedural mucosal disruption. Treatment consisted of vancomycin followed by oral amoxicillin.

The patient also developed epigastric abdominal pain radiating to the back, accompanied by nausea but without vomiting, diarrhea, or fever. Examination revealed abdominal tenderness and pigmented macular lesions on her face, chest, and right leg, corresponding to her previously confirmed LC. Imaging studies showed biliary obstruction with tumefactive sludge, mild biliary dilatation, diffuse hepatic steatosis, hepatosplenomegaly, and gastritis. Endoscopic retrograde cholangiopancreatography was performed to address a distal bile duct stricture. The procedure included sphincterotomy and metal stent placement but was complicated by pancreatitis following the endoscopic retrograde cholangiopancreatography, which resolved with supportive care.

Hematologic evaluations revealed persistent severe pancytopenia. White blood cell counts were less than 0.1 × 10⁹/L, hemoglobin levels were between 6.9 and 8.2 g/dL, and platelet counts were between 8 and 17/µL. Liver function tests showed elevated levels of aspartate aminotransferase (154 U/L), alanine aminotransferase (250 U/L), and alkaline phosphatase (179 U/L). Total bilirubin was 4.8 mg/dL, and direct bilirubin was 2.7 mg/dL. Disseminated intravascular coagulation and hemolysis were ruled out. Table 1 summarizes the hematologic and biochemical findings.

**Table 1:** Summary of the hematologic and biochemical findings These findings are consistent with severe bone marrow suppression. Normal reference ranges were obtained from the Lyndon B. Johnson General Hospital Clinical Laboratory, as per patient laboratory reports [12]. /L - per liter; g/dL - grams per deciliter; /μL - per microliter; U/L - units per liter; mg/dL - milligrams per deciliter

Parameter	Result	Normal range [12]
White blood cell count	<0.1 × 10⁹/L	4.0-11.0 × 10⁹/L
Hemoglobin	6.9-8.2 g/dL	12.0-16.0 g/dL (females)
Platelet count	8-17/μL	150-450 × 10³/μL
Aspartate aminotransferase	154 U/L	10-40 U/L
Alanine aminotransferase	250 U/L	7-56 U/L
Alkaline phosphatase	179 U/L	44-147 U/L
Total bilirubin	4.8 mg/dL	0.1-1.2 mg/dL
Direct bilirubin	2.7 mg/dL	0.0-0.3 mg/dL
Disseminated intravascular coagulation	Ruled out	-
Hemolysis	Ruled out	-

Neutropenia was managed with granulocyte colony-stimulating agents, including tbo-filgrastim and pegfilgrastim-bmtp. Prophylactic antibiotics and antifungals were continued. Valacyclovir was administered to prevent reactivation of herpesviruses. Ciprofloxacin was used to target bacterial infections, and posaconazole provided protection against invasive fungal infections. Famotidine reduced the risk of gastric complications, including bleeding or acid reflux. Ursodiol was prescribed to address liver enzyme abnormalities. Due to prolonged cytopenias, the patient required multiple transfusions. Dose reductions were therefore planned for subsequent chemotherapy cycles.

**Figure 2:**
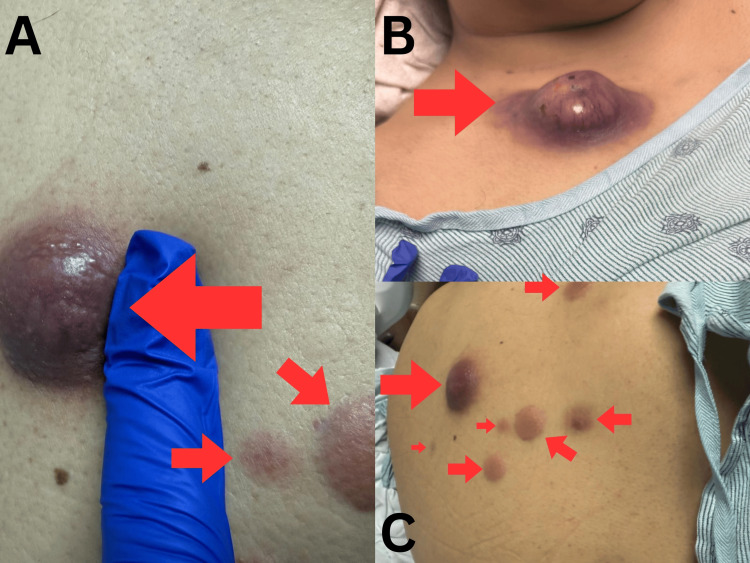
Progression of cutaneous lesions. Note the increase in size of lesions. A finger was placed alongside the nodules to provide a visual scale for size estimation. The lesions have been monitored for changes in size and number, which are indicative of disease progression. The appearance and distribution of the lesions, along with their persistence despite treatment, suggest a potential relapse of acute myeloid leukemia. (A) A close-up view of a large, firm, erythematous nodule with surrounding induration on the right upper back, which is a zoomed-in version of the smaller reddish-brown nodules seen in (C). (B) A rapidly enlarging violaceous nodule with central necrosis on the right upper chest. (C) Multiple smaller reddish-brown nodules forming a linear pattern on the right upper back.

Despite the initial improvement of her condition, skin lesions associated with LC continued to grow in size, as illustrated in Figures 2A-2C. Concerns about progressively growing cutaneous lesions prompted bone marrow biopsy, which confirmed a significant disease relapse with 60% blasts: confirming the progression of AML, with cutaneous manifestations preceding systemic relapse. Three months later, the patient suffered a sudden thalamic hemorrhage with intraventricular extension, resulting in superior and inferior transtentorial herniation, ultimately leading to her death.

This case highlights the aggressive nature of AML with concurrent LC and the potential for cutaneous manifestations to precede systemic relapse, as in this case. Despite an initially favorable response to intensive therapy, the patient's course was complicated by refractory LC, pancytopenia, infectious challenges, and hepatobiliary complications.

## Discussion

LC, a cutaneous manifestation of systemic leukemia, is an uncommon but significant complication. In most instances, it arises concurrently with or subsequent to the diagnosis of a myeloid disorder. Specifically, it occurs concurrently in 25% of cases and after diagnosis in 60% of cases [13]. In a minority of cases (approximately 5%), these cutaneous lesions appear prior to any discernible involvement of the bone marrow or peripheral blood. This phenomenon is recognized as aleukemic LC [13,14]. LC lesions have been reported to have a predilection for the extremities and trunk, which is consistent with our case [15]. As demonstrated in our case, progressively enlarging skin nodules should raise suspicion for systemic AML relapse.

The presence of LC in AML is associated with a poor prognosis, often indicating widespread extramedullary disease. A matched-cohort study demonstrated a substantially increased risk of mortality, likely due to LC serving as a marker of leukemic dissemination and disease aggressiveness. These patients were 2.06 times more likely to die from leukemia and 1.66 times more likely to die from any cause compared to AML patients without LC [16]. The five-year survival rate for patients with LC was significantly lower at 8.6% compared to 28.3% in the matched cohort without LC [16]. LC also highly correlates with sites of additional extramedullary involvement and vasculitis with leukemic cell infiltration [17,18]. This further emphasizes the association of LC with a poor prognosis. Previous studies have also reported LC preceding bone marrow relapse in different subtypes of AML, similar to our case [13,19,20].

While LC is a recognized manifestation of AML, its clinical presentation can mimic various dermatological conditions, necessitating careful differentiation. Infectious skin lesions, such as cellulitis or fungal infections, may present with erythema, induration, and tenderness, whereas inflammatory conditions like cutaneous vasculitis or sarcoidosis can exhibit nodular or plaque-like lesions [21,22]. Unlike these conditions, LC typically manifests as non-tender, violaceous to erythematous nodules or plaques, often without signs of localized infection or systemic inflammatory markers [9,10]. Histopathological evaluation and immunohistochemical staining remain essential for distinguishing LC from other dermatological mimics, ensuring timely diagnosis and appropriate management.

LC highlights the importance of systemic therapy tailored to the specific leukemia subtype. For adult AML, management is guided by patient factors such as age, fitness, genetic mutations, and measurable residual disease status [23]. Fit patients often receive the standard 7+3 induction chemotherapy regimen. This regimen consists of seven days of cytarabine and three days of an anthracycline, such as daunorubicin or idarubicin. Consolidation therapy involves high-dose cytarabine or allogeneic stem cell transplantation, when appropriate [23,24]. Advances in targeted therapies have enhanced treatment precision [25,26]. These include FMS-like tyrosine kinase 3 (FLT-3) inhibitors, such as midostaurin, for FLT3-mutated AML, and isocitrate dehydrogenase (IDH) inhibitors, such as ivosidenib or enasidenib, for IDH-mutated AML. For older or unfit patients, low-intensity regimens combining hypomethylating agents, such as azacitidine or decitabine, with venetoclax offer promising efficacy with reduced toxicity [27]. However, in patients with LC, these regimens may be insufficient to control disease progression, particularly in cases of rapid recurrence or high leukemic burden.

The relapse of AML, including the development of LC, is primarily driven by the persistence of leukemic stem cells that are resistant to conventional treatments. These leukemic stem cells can remain quiescent during chemotherapy, evading the cytotoxic effects of treatment [28]. This allows them to survive and reinitiate the disease upon cessation of therapy. In some cases, such as with LC, relapse may be more apparent in extramedullary sites before the bone marrow shows signs of involvement, as demonstrated in our case [28].

The management of patients with LC presents significant challenges. Our case highlights the aggressive nature of the disease, emphasizing the need for early recognition and prompt initiation of appropriate systemic therapy. Multidisciplinary care and vigilant monitoring are critical in addressing these challenges. The management of relapsed AML with LC requires personalized treatment strategies, timely diagnostic interventions, and comprehensive supportive care. This case also underscores the importance of integrating multidisciplinary approaches to optimize outcomes in patients with complex hematologic malignancies.

A comprehensive diagnostic approach was essential in this case, given the atypical presentation of LC and its role in signaling systemic relapse. The patient initially showed clinical improvement; however, the persistent and progressively enlarging cutaneous lesions warranted further investigation. Serial laboratory assessments, including complete blood counts and peripheral smears, were closely monitored to detect early hematologic changes suggestive of relapse. Contrast-enhanced CT scans were performed to evaluate the extent of extramedullary involvement, while bone marrow biopsy confirmed a resurgence of leukemic blasts. Notably, the cutaneous lesions preceded bone marrow relapse, highlighting the need for heightened clinical vigilance in AML patients with persistent or new-onset dermatologic findings.

The timing of symptom onset and disease recurrence played a crucial role in clinical decision-making. The patient had initially responded to induction therapy with a 7+3 regimen consisting of an anthracycline and low-dose cytarabine, followed by four cycles of high-dose cytarabine for consolidation. Despite this initial therapeutic response, the patient’s clinical course was complicated by refractory LC in the trunk and extremities, persistent pancytopenia with neutropenic fever, and systemic infections. Blood cultures revealed carbapenem-resistant and extended-spectrum beta-lactamase-producing *Enterobacterales*, requiring targeted therapy with cefiderocol. Additionally, *Streptococcus mitis*, likely due to periprocedural mucosal disruption, was later isolated and treated with vancomycin followed by oral amoxicillin. These infectious complications, coupled with progressive disease, ultimately contributed to the patient’s clinical decline, underscoring the complex challenges in managing relapsed AML with extramedullary involvement.

Similar to the life-threatening extramedullary complications seen in other hematologic malignancies, such as spontaneous splenic rupture in chronic myeloid leukemia [29] and central nervous system involvement in acute lymphoblastic leukemia [30], this case illustrates the severity and potential for rapid deterioration associated with extramedullary involvement. These parallels underscore the need for heightened clinical vigilance, as early detection and intervention may significantly alter the disease course and improve patient outcomes. In light of this, further research into novel therapeutic strategies is warranted, including targeted therapies, immunotherapies, allogeneic transplantation, and combinations of existing agents. Specifically, clinical trials investigating approaches tailored to patients with extramedullary manifestations in AML are crucial for advancing treatment and improving survival in this challenging patient population.

While this case provides valuable insights into the aggressive nature of AML with LC and highlights the importance of early recognition and personalized treatment strategies, several limitations should be considered. First, this is a single case report, and the findings may not be generalizable to all patients with AML and LC. Additionally, the lack of detailed molecular and genetic analysis, which could provide further insights into the underlying mechanisms of disease progression and resistance to treatment, is a limitation. Furthermore, the absence of long-term follow-up data limits our ability to assess the long-term outcomes and potential for recurrence or secondary malignancies. Finally, while we provided a comprehensive management approach, there may be alternative treatment regimens that could have been explored, particularly in the context of the patient’s refractory disease.

## Conclusions

In light of the findings in this case, we emphasize that LC in AML is not only associated with a poor prognosis but also often reflects an aggressive disease course. As demonstrated in our patient, cutaneous manifestations may precede systemic relapse, highlighting the importance of close monitoring for early signs of disease progression. Despite the initial improvement of her condition, our patient experienced rapid disease progression, underscoring the need for more effective treatment strategies. This case further illustrates the complexity of managing AML with LC and the critical need for a personalized approach to therapy. Given the aggressive nature of this manifestation and its impact on prognosis, additional research into novel therapeutic approaches, including targeted therapies and immunotherapies, is essential. Such advancements are crucial to improving outcomes for this patient population and addressing the challenges posed by extramedullary relapses in AML.
